# POOR SLEEP COMMON AMONG HOSPICE FAMILY CAREGIVERS AND ASSOCIATED WITH WORSE CAREGIVER HEALTH

**DOI:** 10.1093/geroni/igac059.2555

**Published:** 2022-12-20

**Authors:** Lauren Starr, Karla Washington, Kyle Pitzer, George Demiris, Debra Parker Oliver

**Affiliations:** University of Pennsylvania, Philadelphia, Pennsylvania, United States; Washington University in St. Louis, St. Louis, Missouri, United States; Washington University in St. Louis, St. Louis, Missouri, United States; University of Pennsylvania, Philadelphia, Pennsylvania, United States; Washington University in St. Louis, St. Louis, Missouri, United States

## Abstract

Over 1.2 million hospice family caregivers in the United States are at risk for interrupted, insufficient sleep due to overnight caregiving responsibilities, anxiety or intrusive thoughts, and inadequate caregiving support. Insomnia contributes to health inequities yet is underrecognized and undertreated. The prevalence of insomnia among hospice family caregivers is not well understood. The purpose of this preliminary study was to describe insomnia prevalence among hospice family caregivers and identify factors that differentiate caregivers with sleep difficulties from caregivers without sleep difficulties. This observational study included 57 hospice family caregivers of cancer patients enrolled in a randomized clinical trial for caregivers [NCT02929108]. Results showed 49.1% of hospice family caregivers had subthreshold insomnia to severe clinical insomnia, as measured by the Insomnia Severity Index. Although social determinant of health variables did not differ based on caregiver insomnia status, caregivers with insomnia (5.0, median) self-rated their physical health significantly lower than caregivers without insomnia (8.0, median) (P< 0.001). Directionally, the distance a caregiver lived from a care-recipient also differed by insomnia status, with 70% of caregivers with insomnia co-residing compared to 46% of caregivers without insomnia. Overall, 78.9% of hospice family caregivers had no caregiving support; anxiety and depression were highly prevalent. Clinicians should screen hospice family caregivers for sleep disorders and seek to improve caregiver health by offering sleep interventions tailored to the specific needs of hospice family caregivers and connecting caregivers to health resources for their own wellbeing. Policy makers must expand hospice benefits to include additional caregiver support.

